# Impact of smoking on thyroid gland: dose-related effect of urinary cotinine levels on thyroid function and thyroid autoimmunity

**DOI:** 10.1038/s41598-019-40708-1

**Published:** 2019-03-12

**Authors:** Su-jin Kim, Min Joo Kim, Sang Gab Yoon, Jun Pyo Myong, Hyeong Won Yu, Young Jun Chai, June Young Choi, Kyu Eun Lee

**Affiliations:** 1 Department of Surgery, Seoul National University Hospital and College of Medicine, Seoul, Korea. Cancer Research Institute, Seoul National University College of Medicine, Seoul, Korea. Division of Surgery, Thyroid Center, Seoul National University Cancer Hospital, Seoul, Republic of Korea; 2 Department of Internal Medicine, Seoul National University College of Medicine, Seoul, Republic of Korea; 3 Department of Occupational and Environmental Medicine, Seoul St. Mary’s Hospital, College of Medicine, The Catholic University of Korea, Seoul, Republic of Korea; 4 Department of Surgery, Bundang Seoul National University Hospital, Seoul, Republic of Korea; 5 Department of Surgery, Seoul National University Boramae Medical Center, Seoul, Republic of Korea

## Abstract

Cigarette smoking is believed to affect thyroid function and autoimmune thyroid disease. However, there is little information to analyze their association using objective biomarkers. The aim of this study was to investigate the dose-related effect of smoking on thyroid hormones and thyroid peroxidase antibody (TPO Ab) using urinary cotinine levels and a population-based cohort. The present study used the thyroid hormone and urinary cotinine dataset from sixth Korean National Health and Nutrition Examination Survey in 2014 and 2015, and a total of 4249 participants were included. Dose-response relationships between thyroid hormone (free T4, TSH, and TPO Ab) and urinary cotinine levels were estimated using ANCOVA after adjustment for all variables (age, height, weight, alcohol, exercise, and log- transformed iodine in urine). Urinary cotinine level was negatively correlated with TSH. The estimated coefficients were −0.0711 in males and −0.0941 in females (p < 0.0001). TPO Ab titer was positively correlated with cotinine levels in males (p < 0.0001). Our findings indicated a significant dose-related effect of urinary cotinine level on thyroid function, and thyroid autoimmunity.

## Introduction

Cigarette smoking is believed to affect thyroid function and autoimmune thyroid disease^1^. Exposure to tobacco smoke was previously reported to have variable effects on thyroid function. Several studies that used a questionnaire to assess smoking status have shown that smokers had higher levels of thyroid hormones than non-smokers^2–4^, but results from other studies did not show this effect^5,6^. In addition, a recent study revealed that smokers had lower levels of thyroid peroxidase antibodies (TPO Ab) than non-smokers, suggesting a lower prevalence of autoimmune thyroid disease in smokers than non-smokers^7,8^.

Assessment using a questionnaire has some limitations, such as information bias. There may be a discrepancy between actual and self-reported smoking status. Therefore, objective biomarkers for smoking, such as nicotine, cotinine, and exhaled carbon monoxide, have been investigated and used to validate the smoking status^9^. For example, one report found that serum cotinine was detected in self-reported non-smokers^10^. The study subjects may not recall or may hide their smoking status. A recent publication reported that among women who self-reported as never-smokers, 54.2% were shown to be smokers based on urinary cotinine^11^. Therefore, more objective and quantitative assessment methods to evaluate smoking status are needed.

In previous studies examining the effects of tobacco smoking on thyroid function, a self-reporting questionnaire was used to evaluate smoking status, and the subjects were categorized into active/passive/non-smoker and current/former/never-smoker groups^2–6^. A potential information bias might affect the interpretation of the results. Therefore, the relationship between tobacco smoking and thyroid hormone status should be assessed with an objective validating tool for smoking, such as cotinine levels.

In the present study, we used urinary cotinine level to evaluate the smoking status and analyzed the relationship between cigarette smoking and thyroid hormones or TPO Ab in a population-based cohort.

## Methods

### Study population

The present data were derived from the sixth Korean National Health and Nutrition Examination Survey (KNHANES VI) in 2014 and 2015. The KNHANES is conducted periodically to provide representative national statistics on topics such as nutrition, health behaviors, and examination results among the Korean population by the Korean Centers for Disease Control and Prevention (KCDC). A multi-stage clustered probability analysis was used to select a representative population of non-institutionalized Koreans. The overall participation rates of the KNHANES VI were 78.3%. The thyroid hormone and urinary cotinine levels were derived from the dataset of the KNHANES in 2014 and 2015. The detailed scheme of the KNHANES was described previously^12^. The overall study population for both the thyroid hormone and urinary cotinine measurements was 4357 (2141 in 2014 and 2216 in 2015). Those who were ever diagnosed with thyroid cancer and thyroid disease by doctors or underwent thyroid hormone replacement were excluded (n = 108). A total of 4249 subjects were included in the present study.

### Measurement of thyroid hormone

Free thyroxine (free T4), thyroid-stimulating hormone (TSH), and TPO Ab levels were assessed via blood sampling. Free T4, TSH, and TPO Ab were measured using Roche COBAS 8000 E-602 (Roche, Mannheim, German) with immunoassays. An internal validation was performed monthly. The monthly coefficients of variation were within an acceptable range (for TSH, free T4, and TPO Ab: ≤7%, 7%, and 10%, respectively). External quality assessment was performed by both the College of American Pathologists and the Korean Association of External Quality Assessment Service. The detailed quality control reports were described previously^13,14^.

### Measurement of urinary cotinine

Spot urine samples were obtained by collecting 20–30 mL of mid-stream urine and sealed in a collecting container. The collected urine sample was delivered to the main laboratory and stored at a temperature of 2~8 °C in a refrigerator. Gas chromatography-mass spectrometry (GC-MS) with a Clarus 600/600 T system (Perkin Elmer, Waltham, MA, USA) was used to quantitatively measure urine cotinine. An internal quality control report showed that the coefficients of variation were within 5% or less. An external quality assessment was performed under the German External Quality Assessment Scheme (G-EQUAS). The detailed quality control reports were described elsewhere^13,14^.

Based on the literature^9,15–17^, a cut-off urinary cotinine level of 50 ng/ml or more was used to define cotinine-verified smokers.

### Other measurements

The subjects were classified into 5 groups based on age (those aged <30, 30–39, 40–49, 50–59, and ≥60). Height and weight of the study population were assessed and are shown as cm and kg units, respectively. The health behaviors of the subjects were exercise (weight-bearing exercise over 30 min/days: 0 times per week; 1~2 times per week; 3 or more times per week) and alcohol consumption (less than/more than once per month in the past year). Comorbidities were validated with ‘doctors diagnosed’ on diabetes, dyslipidemia, hypertension, rheumatoid arthritis, and other cancer (gastric cancer, hepatic cancer, colon cancer, breast cancer, cervical cancer (for females), lung cancer). Iodine excretion level was assessed by urinary iodine level (µg/L). Urinary iodine was measured using a Perkin Elmer ICP-MS system (Perkin Elmer, Waltham, MA, USA).

### Statistical analysis

**Figure 1:**
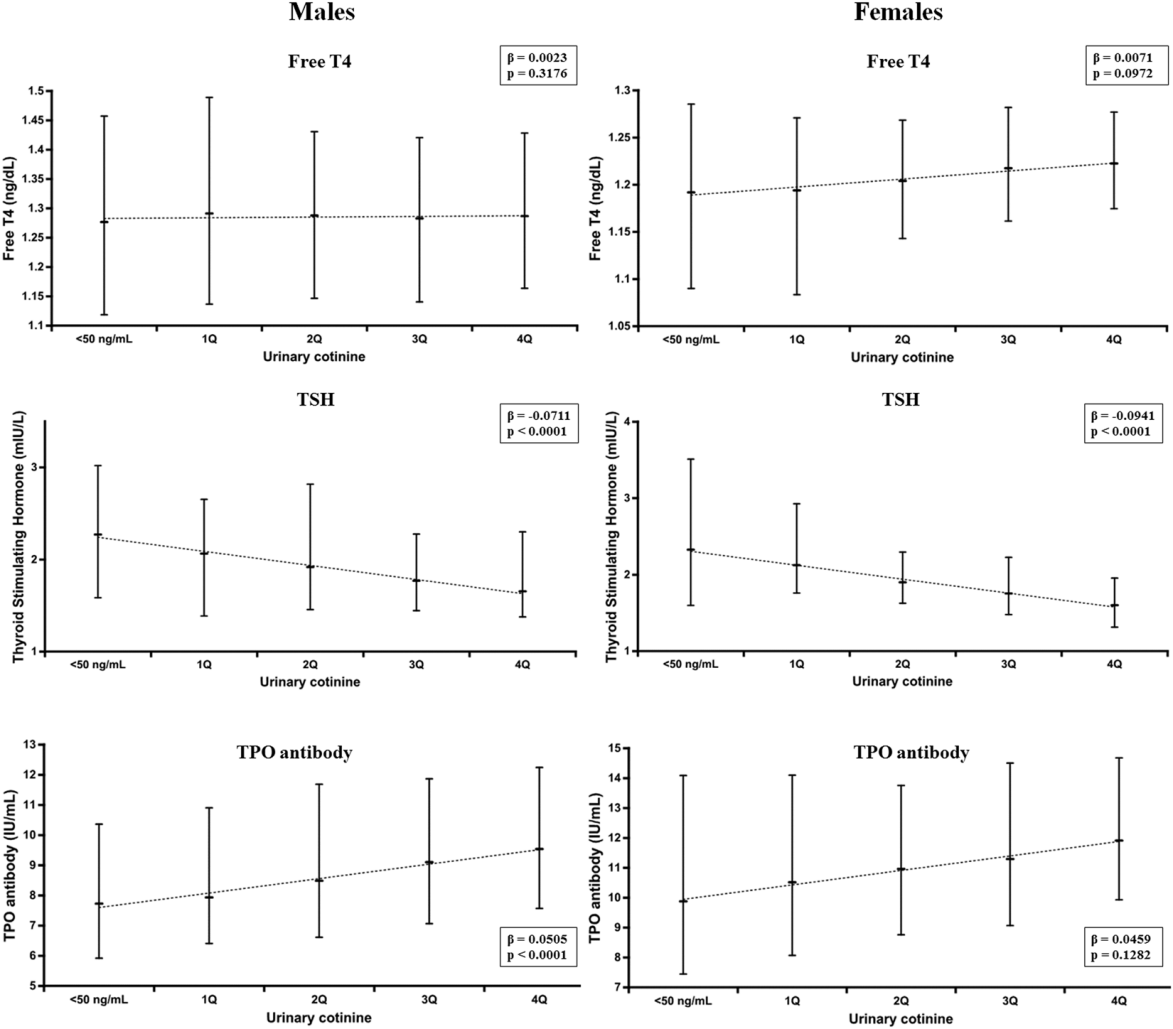
Dose–response relationships among thyroid function, thyroid autoantibodies and urinary cotinine level using ANCOVA methods after adjustment for age, height, weight, health behaviors (alcohol and exercise) and log-transformed urinary iodine by gender.

The distributions of free T4, TSH, and TPO Ab titer in blood and iodine concentration in urine were skewed and log-transformed to estimate the geometric mean (GM) and geometric standard deviation (GSD). Age, height, and weights are shown as the mean and standard deviation. Urinary cotinine level was classified into five groups [currently non-smoker, urinary cotinine (Ucot) < 50 ng/ml; first quartile through fourth quartile among those with Ucot ≥ 50 ng/ml]. Urinary cotinine levels (ng/mL) for males were as follows: first quartile (1Q): 50 ≤ Ucot < 712.540; second quartile (2Q): 712.540 ≤ Ucot < 1238.710; third quartile (3Q): 1238.710 ≤ Ucot < 1822.965; fourth quartile (4Q) 1822.965 ≤ Ucot. Urinary cotinine levels (ng/mL) for females were as follows: 1Q: 50 ≤ Ucot < 442.100; 2Q: 442.100 ≤ Ucot < 810.885; 3Q: 810.885 ≤ Ucot < 1291.985; 4Q 1291.985 ≤ Ucot. A crude trend of log-transformed free T4, TSH, and TPO antibody by urinary cotinine and *p* values for linear trends were estimated using generalized linear models. Dose-response relationships between thyroid hormones (free T4, TSH, and TPO) and urinary cotinine levels were estimated using ANCOVA after adjustment for all variables [age, height, weight, health behaviors (alcohol and exercise) and log-transformed iodine in urine] in Korean males and females. The least squares mean values of the free T4, TSH, and TPO by urinary cotinine levels after ANCOVA analysis are shown with both the upper and lower limits in Fig. 1. All statistical analyses were performed with SAS software (version 9.4 SAS Institute, Inc., Cary, North Carolina, USA).

## Results

Table 1 shows the general characteristics of the study population by gender. The overall mean ages of males and females in the present study were 40.4 years [standard deviation (SD) = 17.7] and 41.1 years (17.4), respectively. The proportions of subjects who participated in weight-bearing exercise over 30 min/day (3 or more times per week) were 28.5% and 14.1% in males and females, respectively. For males, the GMs (GSD) of free T4, TSH, and TPO Ab were 1.28 ng/dL (1.16), 2.12 mIU/L (1.99), and 8.09 IU/mL (2.23), respectively. For females, the GMs (GSD) of free T4, TSH, and TPO Ab were 1.20 ng/dL (1.17), 2.29 mIU/L (2.22), and 9.92 IU/mL (2.94), respectively. The GM (GSD) of urinary cotinine was 508.8 ng/mL (822.2) in males and 94.2 ng/mL (373.5) in females.

**Table 1:** General characteristics (n = 4249).

		Male (n = 2201)		Female (n = 2048)	
		N/Means/Geometric means	SD (%)	N/Means/Geometric means	SD (%)
Age (year)	Mean	*40.4*	*17.7*	*41.1*	*17.4*
	<30	698	31.71	621	30.32
	30–39	356	16.17	340	16.6
	40–49	377	17.13	337	16.46
	50–59	381	17.31	386	18.85
	≥60	389	17.67	364	17.77
Height (cm)		*170.1*	*7.9*	*158.0*	*6.4*
Weight (kg)		*70.0*	*13.5*	*57.5*	*10.1*
Exercise	No	170	7.97	117	5.87
	1~2 per week	1355	63.53	1594	80.02
	3 or more per week	608	28.5	281	14.11
Alcohol consumption (1 or more times per month)					
	No	833	37.85	1255	61.28
	Yes	1368	62.15	793	38.72
Comorbidities	Diabetes (yes)	133	6.04	103	5.03
	Dyslipidemia (yes)	108	8.18	215	10.50
	Hypertension (yes)	346	15.72	264	12.89
	Rheumatoid arthritis (yes)	15	0.68	24	1.17
	Other cancer* (yes)	33	1.50	36	1.76
Urinary Iodine (µg/L)		*345.5*	*3.1*	*338.7*	*3.3*
Free T4 (ng/dL)		*1.28*	*1.16*	*1.20*	*1.17*
TSH (mIU/L)		*2.12*	*1.99*	*2.29*	*2.22*
TPO Antibody Titer (IU/mL)		*8.09*	*2.23*	*9.92*	*2.94*
Urinary Cotinine (ng/mL)		*508.8*	*822.2*	*94.2*	*373.5*
Total		2201	100	2048	100

*One or more for gastric cancer, hepatic cancer, colon cancer, breast cancer, cervical cancer (for females), and lung cancer

Table 2 shows the GMs (GSD) of free T4, TSH, and TPO Ab by the urinary cotinine levels. A negative correlation between TSH and urinary cotinine was observed in both males and females (p for trend <0.0001). However, there was no association between free T4 and urinary cotinine levels. A positive correlation between TPO Ab titer and urinary cotinine levels was observed only in males (p for trend <0.0001).

**Table 2:** Relationship among urinary cotinine, thyroid function, and thyroid autoimmunity.

		Ucot < 50		Ucot ≥ 50								*P* for trend
				1Q		2Q		3Q		4Q		
		GM	GSD	GM	GSD	GM	GSD	GM	GSD	GM	GSD	
Male												
Free T4 (ng/dL)		1.28	1.16	1.26	1.16	1.27	1.13	1.28	1.21	1.31	1.17	0.4858
TSH (mIU/L)		2.30	1.99	2.08	1.80	1.98	1.89	1.80	2.05	1.63	2.00	<0.0001
TPO Antibody Titer (IU/mL)		7.61	2.14	8.49	2.49	9.18	2.48	8.95	2.40	9.12	2.09	<0.0001
Female												
Free T4 (ng/dL)		1.19	1.17	1.24	1.17	1.24	1.24	1.21	1.17	1.19	1.12	0.2229
TSH (mIU/L)		2.34	2.18	2.20	2.69	1.68	2.88	1.79	2.53	1.70	1.83	<0.0001
TPO Antibody Titer (IU/mL)		9.79	2.93	9.65	2.53	14.89	4.07	9.63	2.78	11.83	2.78	0.0873

Urinary cotinine levels (ng/mL) for males were as follows: 1Q: 50 ≤ Ucot < 712.540; 2Q: 712.540 ≤ Ucot < 1238.710; 3Q: 1238.710 ≤ Ucot < 1822.965; 4Q: 1822.965 ≤ Ucot.

Urinary cotinine levels (ng/mL) for females were as follows: 1Q: 50 ≤ Ucot < 442.100; 2Q: 442.100 ≤ Ucot < 810.885; 3Q: 810.885 ≤ Ucot < 1291.985; 4Q: 1291.985 ≤ Ucot.

Figure 1 shows the least squares means and upper/lower limits of free T4, TSH, and TPO Ab by the urinary cotinine levels using ANCOVA methods after adjustment for age, height, weight, health behaviors (alcohol and exercise) and log-transformed urinary iodine. The estimated coefficient for TSH was −0.0711 in males and −0.0941 in females (p < 0.0001). TPO Ab titer increased significantly in males (p < 0.0001). For females, the TPO Ab titer was likely to increase, but the results were not significant (p = 0.1282).

## Discussion

The dose-effect relationships of current level of smoking (negative relationship for TSH; positive relationship for TPO Ab) were shown in the present population-based study.

The purpose of this study was to evaluate the dose-effect relationship between urinary cotinine and thyroid function or TPO Ab using a population-based cohort. Smoke exposure may affect various metabolic and biological processes, including hormone biosynthesis and secretion; interfere with thyroid hormone release, binding, transport, storage, and clearance; and be associated with adverse effects on the thyroid, resulting in changes in circulating hormone concentrations^18^. In addition, tobacco smoke may also play a role in thyroid autoimmunity. Many studies have reported a significant effect of smoking on Graves’ hyperthyroidism and particularly on Graves’ orbitopathy^19–21^. However, the effect of smoking in Hashimoto’s thyroiditis is not as well established as in Graves’ disease. We found that urinary cotinine level was negatively correlated with TSH level in both males and females and positively correlated with TPO Ab level in males.

Few studies have examined the effect of smoking status on thyroid function using serum cotinine level to evaluate smoking status^7,18^. Soldin *et al*. measured serum cotinine level and categorized subjects into active smokers/passive smokers/non-smokers in a population of 237 non-pregnant women^18^. Active smokers showed decreased TSH and T4 levels. However, the researchers did not analyze the dose relationship between cigarette smoking and thyroid function. Belin *et al*. used serum cotinine level as a continuous variable to analyze the relationship with thyroid function in 15592 subjects from the Third National Health and Nutrition Examination Survey III (United States 1988–1994)^7^. They found that every 10 ng/mL increase in serum cotinine, the odds of having TSH levels were decreased by 1.4% after adjustment for age, gender, race, and urinary iodine status. A dose-dependent relationship between serum cotinine-validated smoking status (active/passive/non-smoking) and serum TSH was observed. Consistent with previous studies, we found that urinary cotinine level was negatively associated with TSH level after controlling for age, height, weight, health behaviors (alcohol consumption, and exercise), and urinary iodine level. To our knowledge, the present study is first report to reveal the dose-related effect of urinary cotinine level on TSH level. There are several possible underlying mechanisms. One may involve the stimulatory effects of smoking on the thyroid gland via increased serum thyroxine-binding globulin and T3 concentrations and decreased serum TSH levels^4,22^. Another mechanism is alterations in iodide transport; smoking may be associated with altered thyroid autoimmunity, suggesting that smoke interferes with iodide transport and organification^5^.

Cigarette smoking has been linked to the development of many autoimmune disease, including rheumatoid arthritis, systemic lupus erythematosus, multiple sclerosis, Graves’ disease, and primary biliary cirrhosis^23–25^. However, the effect of cigarette smoking on Hashimoto’s thyroiditis has not been fully elucidated^26^. Several studies have reported that cigarette smoking is associated with a decreased prevalence of TPO Ab^27,28^, but Cho *et al*. reported no significant association between these parameters^29^. In contrast to previous studies^7,29^. we found a positive trend between urinary cotinine level and high levels of TPO Ab (p < 0.001 in males, p = 0.0873 in females). Most previous studies identified smokers using questionnaires, and the presence of thyroid autoimmunity was defined with various cut-off values of TPO Ab. Belin *et al*. classified smokers based on self-reported questionnaires, confirmed the findings with urinary cotinine values higher than 15 IU/mL, and defined the presence of TPO Ab with a value of 0.5 IU/mL. Cho *et al*. classified smokers based on self-reported questionnaires, and the presence of TPO Ab was defined with a value of 0.3 kU/L^29^. In the present study, a non-smoker was defined with a value less than 50 ng/mL, the severity of smoking was categorized into four quartile groups (1^st^ Q-4^th^ Q), and we used the continuous variable of TPO Ab to evaluate the relationship between cigarette smoking and thyroid autoimmunity. To validate the finding in the present study that TPO Ab is increased in smokers, we analyzed the relationship based on the categories defined by Belin *et al*.^7^ and found that TPO Ab titer was significantly increased in males (p < 0.0001, estimated coefficient = 0.049). For females, the TPO Ab titer was likely to increase; however, the results were not significant (p = 0.2027, estimated coefficient = 0.2027). However, we could not explain the exact reason for different impact of smoking on TPO Ab according to gender, we postulate there are possibility the impact of smoking on female is less small comparing that on men, because Hashimoto’s thyroiditis occurs predominatly in females than in males.

There are several factors that affect the relationship between cigarette smoking and TPO Ab. Hashimoto’s thyroiditis typically occurs between the ages of 30 and 50 and is much more common in women than men, which influence the TPO Ab level^30^. There was a high proportion of young smokers in the current smoker group. Therefore, adjustment for age and gender was needed for accurate analyses. In addition, there may be hidden smokers in the female group; thus, hidden smokers could be categorized in non-smokers with high TPO Ab levels. Objective tools to define smokers and the severity of smoking were important for the present study. Other factors may include differences in iodine intake compared with that of previous studies, which may influence the relationship between smoking and thyroid autoimmunity.

One of the strengths of the present study was the use of urinary cotinine as an objective method to evaluate smoking status quantitatively instead of a self-reporting questionnaire. A categorical classification of smoking status using a self-reported questionnaire is not quantitative. Even if a quantitative analysis using pack-years is performed, it may be inaccurate because it relies on memory. In addition, smoking may be under-reported in Korean women because of the traditional Korean cultural background, which stigmatizes smoking in women^11^. Several biomarkers for tobacco smoking (nicotine, cotinine, and exhaled carbon monoxide) have been used to validate the smoking status^31,32^. Plasma nicotine is the most accurate method for validating smoking status; however, it is difficult to use in the national survey research due to its short half-life (2–3 hr). Therefore, cotinine, a metabolite of nicotine, is appropriate in large-scale nationwide research.

Although there is a relatively large amount of data on the influence of cigarette smoking on the thyroid gland, this influence is exerted through different mechanisms and modified by several factors, such as age, sex, ethnicity and iodine status. Although we did not identify the mechanism underlying the relationship between smoking and thyroid function or thyroid autoimmunity, the present study was based on a large population-based study, and the analysis of the dose-effect relationship among cigarette smoking, thyroid function, and autoimmunity was adjusted for age, height, weight, exercise, alcohol consumption, and urinary iodine.

In conclusion, we found that cigarette smoking is associated with decreased TSH levels in both males and females and increased TPO Ab level in male subjects in the general noninstitutionalized Korean population evaluated in the KHANES VI. The present study provides useful information for public health and shows that smoking has significant effects on thyroid function and thyroid autoimmunity.
